# Effect of thoracic epidural anesthesia on heart rate variability in a porcine model

**DOI:** 10.14814/phy2.13116

**Published:** 2017-04-11

**Authors:** Arjun Mahajan, Tatsuo Takamiya, Peyman Benharash, Wei Zhou

**Affiliations:** ^1^ University of California BerkleyBerkleyCalifornia; ^2^ Department of AnesthesiologyUniversity of California Los AngelesLos AngelesCalifornia; ^3^ Division of Cardiac SurgeryUniversity of California Los AngelesLos AngelesCalifornia

**Keywords:** Activation recovery interval, autonomic nerve system, LF/HF, power spectrum

## Abstract

Heart rate variability (HRV) is increasingly recognized as a means of evaluating autonomic tone. Thoracic epidural anesthesia (TEA) has been previously demonstrated to suppress the electrical storms in patients. However, the effect of TEA on HRV during sympathoexcitation remains unknown. In this study, we aimed to determine the effects of TEA on HRV response to left stellate ganglion stimulation (LSS) in a porcine model. In 12 anesthetized pigs after insertion of an epidural catheter to T1 level, a median sternotomy was performed to expose the heart and the left stellate ganglion. A 56‐electrode sock was used for obtaining epicardial activation recovery interval (ARI). Animal received LSS at 4 Hz for 30 sec. After 30 min of bupivacaine epidural injection, LSS was performed in the same way as the baseline condition. LSS significantly increased low‐frequency normalized units (LF: 44.9 ± 6.7 vs. 13.6 ± 3.1 msec^2^ baseline, *P* < 0.05) and decreased high‐frequency normalized units (HF: 11.5 ± 4.6 vs. 41.9 ± 5.1 msec^2^ baseline, *P* < 0.05). As a result, LF/HF significantly increased from 0.3 ± 0.2 to 3.9 ± 1.4 during LSS. TEA significantly attenuated the LF/HF from 3.9 ± 1.4 to 1.6 ± 0.8 with increased HF components from 11.5 ± 4.6 to 26.5 ± 3.2 msec^2^. LF component significantly correlates with global ARI (*r* = −0.81) and dispersion of repolarization (*r* = 0.85). HRV can precisely reflect the cardiac autonomic tone and TEA modulates the HRV by enhancing the HF components probably through a parasympathetic nerve system.

## Introduction

Imbalances in the activity of the sympathetic and parasympathetic nervous systems have been strongly associated with sudden cardiac death (Stein et al. 1999; Vaseghi and Shivkumar 2008). Heart rate variability (HRV) is increasingly recognized as a means of evaluating cardiac autonomic tone (Bailey et al. 1996; Stein et al. 1994). The low‐ frequency (LF) oscillations represent sympathetic while the high‐frequency (HF) component represents parasympathetic nerve activity. Therefore, the ratio of LF to HF can reflect the balance of autonomic nervous system (Malliani et al. 1991).

The sympathetic nervous system plays an important role in arrhythmogenesis (Vaseghi and Shivkumar 2008). Blockade of direct sympathetic stimulation via beta blockers reduces the risk of sudden cardiac death. Sympathectomy reduces ventricular arrhythmic episodes in both animal and human studies (Hofferberth et al. 2014; Kadowaki and Levett 1986; Odero et al. 2010; Zipes 1970). Spinal cord forms afferent and efferent arms and plays an important role in cardiovascular regulation (Armour 2004; Longhurst 2003). Modulation of the spinal cord such as spinal cord stimulation or thoracic epidural anesthesia (TEA) has been shown to decrease arrhythmias in patients (Blomberg and Ricksten 1988; Bourke et al. 2010; Cardinal et al. 2004).

We have previously demonstrated that stellate ganglion stimulation shortens activation recovery intervals (ARI), a surrogate of action potentials, and increases the ARI dispersions (Ajijola et al. 2013; Vaseghi et al. 2012a,b). However, parameters of HRV have not been validated against ARI under such circumstances.

In this study, we aimed to evaluate changes in HRV against ARI differences under conditions of stellate ganglion stimulation and thoracic epidural anesthesia in a porcine model.

## Methods

### Ethics statement and animal model preparation

All animal experimental studies were performed in accordance with the guidelines of the University of California Institutional Animal Care and Use Committee and the National Institutes of Health Guide for the Care and Use of Laboratory Animals (NIH Pub. No. 85‐23, Revised 1996).

Twelve Yorkshire pigs (weighing 38–50 kg, five males and seven females) were sedated with telazol (8–10 mg/kg, intramuscular), intubated and mechanically ventilated. General anesthesia consisted of isoflurane (2–3%) during surgical preparation. Surface 12‐lead ECG was monitored using a Prucka‐CardioLab recording system (GE Healthcare, Fairfield, CT). The femoral artery and vein were catheterized for monitoring of arterial blood pressure, intravenous saline infusion (10 mL/kg), and drug administration. In order to maintain acid‐base equilibrium, arterial blood gasses were checked hourly and triggered adjustments to ventilation or administration of IV sodium bicarbonate administration to maintain a pH of 7.35–7.42. The animal was positioned in the right lateral decubitus position and using a small midline incision at the T‐7/8 level, an 18‐gage Touhy needle was inserted into the epidural space using the loss of resistance technique. At the end of the experiment, a laminectomy was performed to confirm the position of the TEA catheter and the spread of the anesthetic mixed with methylene blue.

A median sternotomy was subsequently performed in the supine position. The left stellate ganglia were isolated and the pericardium was opened to expose the heart. Following the completion of surgical preparation, general anesthesia was transitioned to intravenous *α*‐chloralose (50 mg/kg initial bolus followed by a 20 mg/kg/h continuous infusion), and animals were allowed to stabilize for 1 h.

### Hemodynamic assessment:

To measure left ventricular (LV) pressure throughout the experiment, a conductance pressure‐volume catheter (5 Fr) was inserted into the LV via the left common carotid artery and connected to a MPVS Ultra Pressure Volume Loop System (Millar Instruments, Houston, TX). Catheter placement was confirmed by using ultrasound guidance to obtain appropriate pressure‐volume loops.

### Left Stellate ganglion stimulation

Left stellate ganglion stimulation (LSS) was performed using bipolar needle electrodes. Square stimulation pulses 4 ms in duration and 4 Hz in frequency via a Grass S88 Stimulator (Grass Co., Warwick, RI). Stimulation threshold was defined as the stimulation current strength to elicit a 10% increase of left ventricular end systolic pressure (LVESP). Thereafter, stimulus intensity was increased to twice the threshold for all subsequent LSS.

### ARI recording and analysis

**Figure 1:**
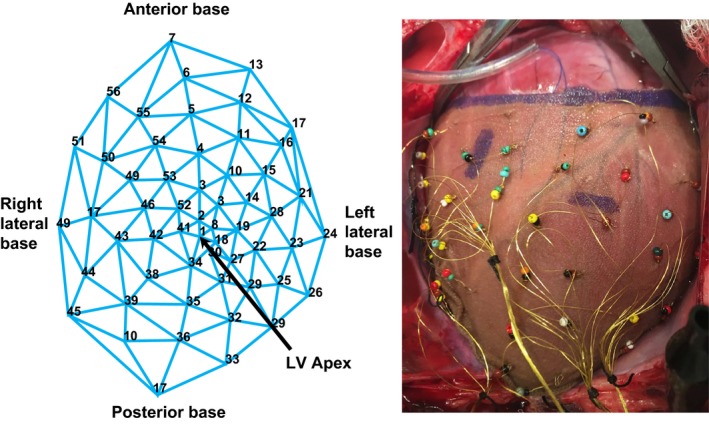
Schematic representation of the 56‐electrode sock including relative positions of the right ventricle and left ventricle.

**Figure 2:**
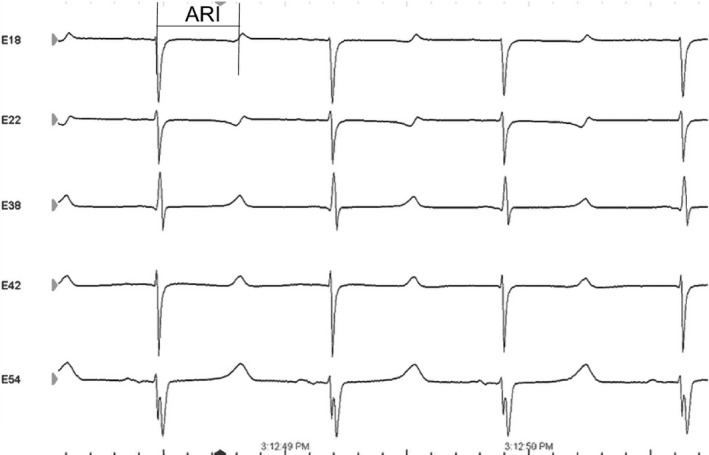
Activation recovery interval (ARI) measurements. A representative sample of 5 electrograms at baseline. ARI represents the time between the most negative dV/dt of the activation wave and the most positive dV/dt of the repolarization wave.

A custom 56‐electrode epicardial sock was placed around the heart to acquire unipolar ventricular epicardial electrograms recorded by a PruckaCardioLab system (GE Healthcare, Fairfield, CT) (Fig. 1). Activation recovery intervals (ARIs) were analyzed by using the customized software iScalDyn (University of Utah, Salt Lake City, UT) as we previously described (Ajijola et al. 2013; Vaseghi et al. 2012a). Briefly, using the onset of the QRS complex, activation time (AT) was measured as the minimal dV/dt, and repolarization time (RT) as the maximal dV/dt in the T wave. ARIs were then calculated by subtracting the AT from the RT (Fig. 2). ARI has been previously demonstrated to correlate well with local action potential duration (APD). Whole heart epicardial dispersion of repolarization (DOR) was calculated using the variance of all 56‐electrode ARIs.

### Heart rate variability analysis

The ECG signal was continuously recorded by a MPVS Ultra Pressure Volume Loop System (Millar Instruments, Houston, TX). Power spectral analysis of the RR‐interval variability was performed using Lab Chart 8 (AD Instruments). The LF component was calculated as the power within the frequency of 0.04–0.15 Hz, and the HF component as the power within the frequency range of 0.15–0.4 Hz. The power of the LF and HF components were calculated separately for the baseline, LSS, and TEA with LSS periods.

### Experimental protocol

After completion of baseline measurements, LSS was performed for 30 seconds followed by a 30‐min resting interval in order to have hemodynamics and ventricular ARI recover to the baseline condition. Local anesthesia with 0.25% bupivacaine (0.7 mg/kg) with 0.1 mL methylene blue was injected via an epidural catheter within 1 min. After 30 min of bupivacaine injection, LSS was performed in the same way as the baseline condition.

### Statistical analysis

Data were reported as mean ± standard error (SEM). Analysis was performed using SigmaPlot (version 11). One‐way repeated ANOVA followed by Tukey post hoc test was performed to compare ARI and hemodynamic changes among four conditions (1) baseline, (2) LSS, (3) baseline TEA, and (4) LSS + TEA. Differences were considered statistically significant with *P* < 0.05.

## Results

### Hemodynamic response to LSS and TEA

**Figure 3:**
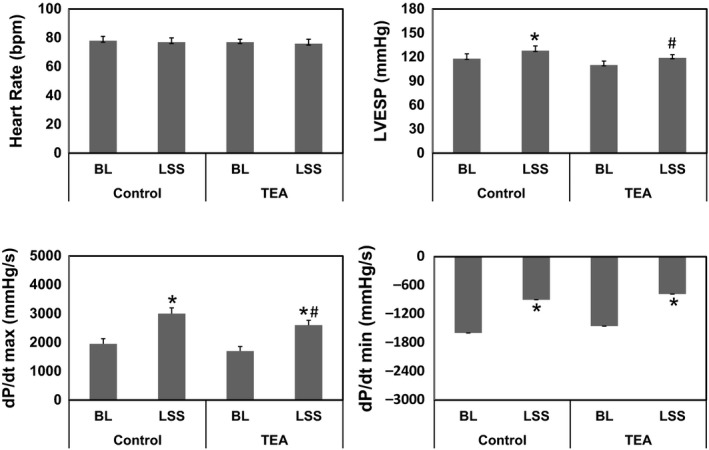
Hemodynamic response to TEA. *P < 0.05, Left stellate ganglion stimulation (LSS) versus baseline (BL); #*P* < 0.05, TEA versus control. TEA, Thoracic epidural anesthesia.

LSS significantly increased LVESP from 115 ± 7 to 127 ± 9 mmHg and dp/dt_max_ from 2050 ± 110 to 3012 ± 145 mmHg/sec (*P* < 0.01). However, heart rate did not change significantly (75 ± 6 vs. 74 ± 5 bpm, *P* > 0.05). TEA did not alter LVESP and dp/dtmax at baseline but significantly attenuated the pressor response to LSS (Fig. 3).

### HRV response to LSS and TEA

**Figure 4:**
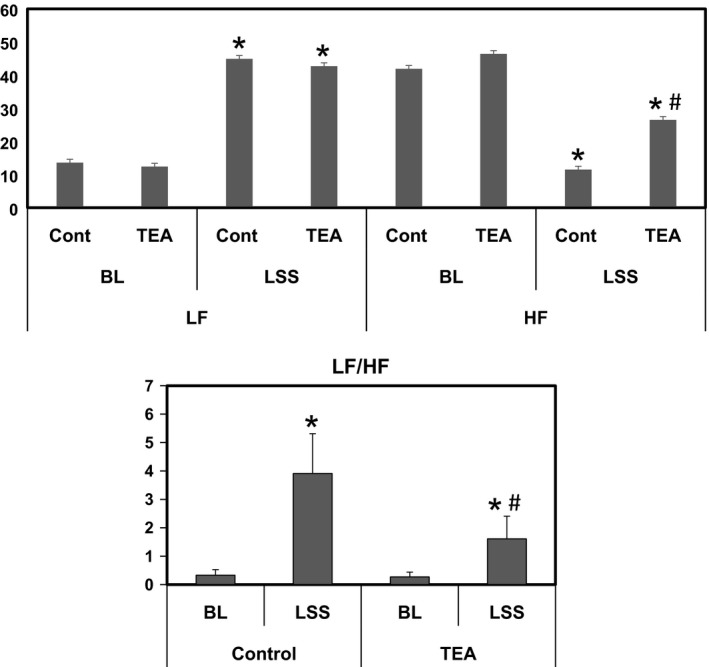
Heart rate variability response to left stellate ganglion stimulation (LSS) with and without TEA. * *P* < 0.05, LSS versus baseline (BL); # *P* < 0.05, TEA versus control. TEA, Thoracic epidural anesthesia; LSS left stellate ganglion stimulation.

Figure 4 shows that LSS significantly increased LF component from 13.6 ± 3 to 44.9 ± 6.7 (msec^2^) and decreased HF from 41.9 ± 5 to 11.5 ± 4.6 (msec^2^) (*P* < 0.05). As a result, LF/HF significantly increased from 0.3 ± 0.2 to 3.9 ± 1.4 during LSS (*P* < 0.05). When compared to control, TEA significantly attenuated the increase in LF/HF (1.6 ± 0.8 vs. 3.9 ± 1.4, *P* < 0.05) during LSS with higher HF components (26.5 ± 3.2 vs.11.5 ± 4.6 msec^2^)

### Correlation between HRV components and ARI parameter during LSS

**Figure 5:**
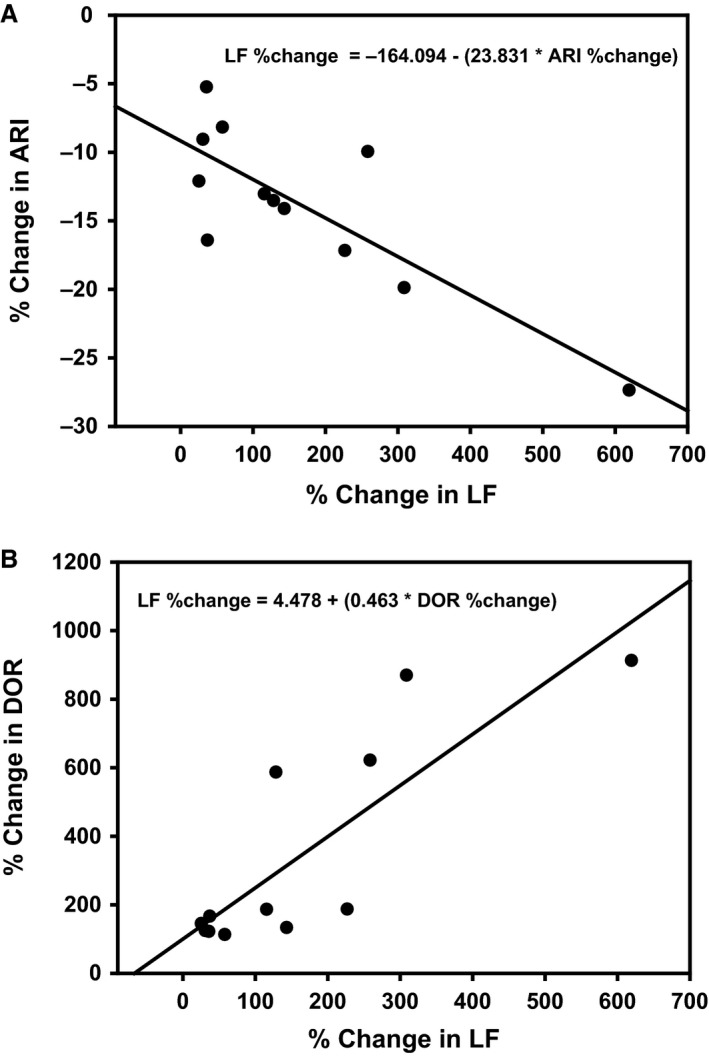
Correlation between low‐frequency component (LF) of heart rate variability and activation recovery interval (ARI) and DOR (dispersion of repolarization).

LSS significantly shortened ARI from 423 ± 18 to 387 ± 21 msec and increased the ARI dispersion from 501 ± 26 to 1753 ± 56 msec^2^ (*P* < 0.05). Table 1 and Figure 5 show that percentage change from baseline during LSS in LF component closely correlates with the percentage change in ARI (*r* = −0.81, *P* < 0.001) and ARI dispersions (*r* = 0.85, *P* < 0.001). However, the percentage change in HF and LF/HF do not have a significant correlation with changes in ARI and DOR during LSS.

**Table 1:** Correlation between HRV Components and ARI/DOR

	ARI	DOR
LF	−0.81 *P* < 0.001	0.86 *P* < 0.001
HF	0.37 *P* = 0.17	−0.38 *P* = 0.16
LF/HF	0.38 *P* = 0.26	0.29 *P* = 0.30

HRV, heart rate variability; ARI, activation recovery interval; DOR, dispersion of repolarization; LF, low frequency; HF, high frequency.

## Discussion

In this study of cardiac autonomic activity in a porcine model, we have found that (1) LF component of HRV significantly correlates with ARI and ARI dispersions; (2) LF/HF significantly increased during LSS while TEA significantly attenuated the increased LF/HF with increased HF components.

### Validation of HRV against ARI during sympathetic efferent stimulation

Activation of the sympathetic nervous system has been associated with a poor prognosis in patients with cardiac disease (Cohn et al. 1984; Vaseghi and Shivkumar 2008). The techniques available to assess sympathetic activation include measurement of plasma catecholamines, sympathetic nerve recordings, and frequency domain analysis of HRV (Stein et al. 1994). Because it is noninvasive and relatively easy to perform, HRV analysis has been widely used in both clinical and experimental studies.

The use of LF for sympathetic activity remains controversial as LF component represents a mixture of sympathetic, parasympathetic and other influences (Billman 2013). However, we have previously validated ARI shortening with left stellate ganglion stimulation on numerous occasions (Ajijola et al. 2012, 2013; Vaseghi et al. 2012a,b). The present work attempts to show associations between an increase in the LF component with ARI shortening induced by sympathetic stimulation.

Left stellate ganglion stimulation (LSS) is known to be arrhythmogenic, with associated increases in early afterdepolarizations, delayed afterdepolarization, overall myocardial dispersion of repolarization (DOR), and an increased incidence of ventricular fibrillation at baseline and during myocardial ischemia (Ajijola et al. 2012). In a porcine model, we have previously demonstrated that LSS significantly shortened ARI and increased DOR as well as interstitial norepinephrine level of the left ventricle, indicating cardiac sympatho‐excitation (Ajijola et al. 2013; Vaseghi et al. 2012b). ARI has been shown to be a reliable a surrogate for APD by use of simultaneously acquired intracellular recordings and monophasic APD recordings and has been validated in both animal models and humans (Chinushi et al. 2001; Haws and Lux 1990; Millar et al. 1985; Yue et al. 2004). This study shows that LF component of HRV significantly correlates with ARI and DOR during LSS, suggesting that spectral analysis of HRV can predict the cardiac neuronal activity by noninvasive measurement.

As shown by Fujiki et al. (1999), the right stellate ganglion directly affects the sinus node and heart rate variability. The goal of our study was to investigate the effect of sympatho‐excitation at the level of the heart and evaluate the autonomic neural control of heart rate/variability. Right‐sided stimulation as shown by our group would have a direct impact on heart rate and therefore not highlight the central component of control (Vaseghi et al. 2012b; Zhou et al. 2013).

### Effect of TEA on HRV during sympathoexcitation

For central control of efferent sympathetic output, the spinal cord represents a major integrative nexus point from afferent neuronal transmission to the heart. Modulation at the level of the thoracic spinal cord has been demonstrated to be an important therapeutic avenue for central control of cardiac arrhythmias. For example, Spinal cord stimulation reduces the incidence of angina pectoris in humans (Andersen et al. 1994) and TEA has been shown to reduce ventricular arrhythmogenesis (Blomberg and Ricksten 1988; Bourke et al. 2010).

However, the effect of TEA on the balance of autonomic nerves system assessed by HRV has not been studied. In this study, we have shown that LSS increased LF/HF while TEA suppressed it. Additionally, TEA increased the HF component. These results suggest that TEA may have sympathoinhibitory effect by enhancing parasympathetic nerve system. Future studies of this particular finding are warranted.

## Clinical Implications

The importance of autonomic tone in cardiac disease has become widely appreciated. Because the presence of HRV is clearly due to autonomic effects on the heart and because its measurement is fairly easy to perform noninvasively, the use of HRV as an index of sympathovagal balance has increased. This study demonstrates that LF components are closely correlated with ARI and ARI dispersions indicating that HRV may serve as a noninvasive measure of cardiac autonomic tone. TEA affects the HRV by enhancing the HF components probably through modulation of the parasympathetic nerve system. These findings provide mechanistic insights on treatment of cardiovascular diseases by neuromodulation therapy.

## Study Limitations

This study was conducted in anesthetized healthy animals. It is possible that the anesthesia may influence the HRV compared to conscious state, and chronic disease condition may also affect the effect of TEA on HRV. Changes in the autonomic state may have secondary effects on other factors such as respiration, which also may affect these measurements.

## Conclusions

HRV can precisely reflect the cardiac autonomic tone and TEA modulates the HRV by enhancing the HF components probably through the parasympathetic nerve system.

## Conflict of Interest

None declared.
